# A Psychometric Evaluation of the Hypoglycemia Problem-Solving Scale (HPSS) in Turkish Older Adults with Diabetes

**DOI:** 10.3390/healthcare13090997

**Published:** 2025-04-25

**Authors:** Merve Dervişoğlu, Dilek Büyükkaya Besen, Merve Günbaş, Mehtap Ertaş, Barış Emekdaş

**Affiliations:** 1 Internal Medicine Nursing Doctorate Program, Institute of Health Sciences, Dokuz Eylul University, 35390 Izmir, Türkiye; 2 Internal Medicine Nursing, Faculty of Nursing, Dokuz Eylul University, 35340 Izmir, Türkiye; 3 Diabetes Education Unit, Çiğli Training and Research Hospital, İzmir Bakırçay University, 35665 Izmir, Türkiye; 4 Internal Medicine Clinic, Çiğli Training and Research Hospital, İzmir Bakırçay University, 35665 Izmir, Türkiye

**Keywords:** diabetes mellitus, problem solving, hypoglycemia, psychometrics

## Abstract

**Introduction:** Hypoglycemia is a significant complication in diabetes management and presents an even greater risk for older adults. These individuals are particularly vulnerable to hypoglycemic episodes, which can result in serious health consequences. The Hypoglycemia Problem-Solving Scale (HPSS) evaluates problem-solving skills related to hypoglycemia; however, it has not yet been validated in Türkiye. This study aimed to evaluate the validity and reliability of the Turkish adaptation of the HPSS. **Materials and Methods**: A descriptive, methodological, and cross-sectional study was conducted with 623 older adults (aged 65 and above) diagnosed with Type 2 diabetes and with a history of hypoglycemic episodes. The HPSS was adapted into Turkish, and its psychometric properties were assessed through content validity, Exploratory Factor Analysis (EFA), Confirmatory Factor Analysis (CFA), and reliability analyses. **Results**: The mean age of participants was 72 ± 5.5 years. Expert agreement on the items was high (Kendall’s W = 0.83, *p* < 0.05), and all items had a Content Validity Ratio (CVR) above 0.56. The overall Content Validity Index (CVI) was 0.97. Exploratory Factor Analysis (EFA) revealed seven factors explaining 74.22% of the total variance. The Kaiser–Meyer–Olkin (KMO) value was 0.85, and Bartlett’s test of sphericity was significant (χ^2^ = 7590.85, *p* < 0.001). Confirmatory Factor Analysis (CFA) demonstrated acceptable model fit (CFI = 0.952; χ^2^/df = 2.536). The scale demonstrated high internal consistency (Cronbach’s alpha = 0.88) and excellent test–retest reliability (r = 0.99, *p* < 0.001). **Discussion/Conclusions**: The Turkish version of the Hypoglycemia Problem-Solving Scale is a valid and reliable instrument for evaluating problem-solving skills related to hypoglycemia in older adults with diabetes. It can be effectively utilized in clinical practice to support better hypoglycemia management and improve overall diabetes care.

## 1. Introduction

Diabetes is a rapidly growing global health issue that poses significant challenges both in clinical management and public health. In 2021, the global prevalence of diabetes among individuals aged 20–79 was estimated to be 10.5%, and this is projected to rise to 12.2% by 2045, potentially affecting approximately 783.2 million people [1,2,3]. Beyond its direct effects on morbidity and mortality, diabetes significantly reduces the quality of life of individuals due to the complexity of its management and imposes a substantial economic burden on health systems. In fact, global healthcare expenditures related to diabetes were reported to be USD 966 billion in 2021, with expectations to reach USD 1054 billion by 2045 [1,2,3,4].

Type 2 diabetes (T2DM), which accounts for the majority of diabetes cases, has aging as a significant risk factor in its development. The pathogenesis of T2DM is closely linked to the aging process, with age-related insulin resistance and progressive β-cell dysfunction being primary causes of hyperglycemia. Although the changes in insulin signaling pathways associated with aging are relatively mild, the changes in body composition, such as increased visceral fat, decreased muscle mass and function (sarcopenia), physical inactivity, and inadequate nutrition, accelerate this process. Additionally, ectopic fat deposition in tissues like the liver, skeletal muscles, and heart also negatively affects insulin sensitivity, increasing the risk of T2DM development [5]. One of the biggest challenges in managing Type 2 diabetes in older individuals is minimizing the risk of hypoglycemia while maintaining optimal glycemic control. In particular, hypoglycemia, especially in individuals using insulin or insulin secretagogues, is a significant clinical concern and is considered one of the main barriers to achieving glycemic targets [1,2,4,5]. Aging reduces the protective physiological mechanisms against hypoglycemia, which increases both the frequency and severity of hypoglycemic events in older adults. In a study by Gehlaut et al. (2015), at least one hypoglycemic episode was detected in 49.1% of Type 2 diabetes patients during a 5-day continuous glucose-monitoring period, with 75% of these episodes being asymptomatic [6]. The failure to recognize symptoms of hypoglycemia leads to a gradual reduction in awareness, increasing the risk of severe hypoglycemia [7,8,9]. Hypoglycemia can cause not only fluctuations in glucose levels but also cardiovascular events, life-threatening arrhythmias, and cognitive function impairments. The literature suggests a bidirectional relationship between hypoglycemia and dementia [9,10]. The decline in cognitive and functional abilities makes it harder for older adults to recognize and appropriately manage hypoglycemia, increasing the risk of complications such as falls, fractures, seizures, coma, and cognitive decline [7,8,9]. The effects of hypoglycemia are not limited to physical health but also have significant psychological, social, and economic consequences. Severe and recurrent hypoglycemic episodes increase emergency-room visits and hospitalization rates, lead to long-term complications, raise healthcare costs, and negatively affect the quality of life [4,5,6,7,8,9,10].

Given these reasons, it is essential to assess and improve hypoglycemia-related problem-solving skills in older individuals [11,12]. Problem-solving skills are a key component of diabetes self-management and play a critical role in reducing hypoglycemia-related risks [12,13,14,15,16,17]. Improving hypoglycemia problem-solving skills not only enhances HbA1c levels but also reduces the frequency of severe hypoglycemic episodes [13,16]. However, despite the importance of problem-solving skills in hypoglycemia management, there is currently no valid and reliable measurement tool available to assess these skills in older diabetic individuals in Türkiye. The Hypoglycemia Problem-Solving Scale (HPSS), developed by Wu et al. (2016), is a comprehensive 24-item scale that evaluates strategies for coping with hypoglycemia and demonstrates strong psychometric properties [15]. The HPSS allows for the identification of factors contributing to hypoglycemia and provides detailed insights into the skills that individuals need to develop to address these issues. The subscales of the scale highlight individual deficiencies in preventing, recognizing, and responding to hypoglycemia, facilitating the planning of targeted interventions. In this context, the HPSS not only evaluates an individual’s capacity for problem solving in relation to hypoglycemia but also encourages the active participation of the individual, their family, and caregivers in this process [13,15,16,18]. The aim of this study is to adapt the Hypoglycemia Problem-Solving Scale (HPSS) into Turkish and evaluate its validity and reliability among older diabetic individuals in Türkiye, offering a qualified tool for measuring hypoglycemia problem-solving skills.

## 2. Materials and Methods

### 2.1. Design

The study was conducted using a descriptive, methodological, and cross-sectional research design to examine the psychometric properties of the HPSS. The research was conducted in accordance with guidelines for the development and validation of scales in the healthcare field, including language validity, content validity, construct validity, and reliability assessment [19].

### 2.2. Language and Content Validity

In the cultural adaptation process of the Hypoglycemia Problem-Solving Scale, a team of four translators was involved in the Turkish translation. Three of the translators were healthcare professionals with expertise in diabetes care, proficient in both the source and target languages, while the fourth translator was a professional translator without healthcare experience. This team ensured the accurate translation of medical terminology and the preservation of the meaning of each item in the target language. After the initial translations were completed, the scale was sent for back-translation to two other translators, proficient in both languages and cultures, with experience in healthcare. During the back-translation process, the researchers compared the original scale with the back-translations to ensure linguistic and cultural accuracy. This step helped identify discrepancies and made it possible to make necessary adjustments to preserve meaning. For example, certain medical terms and expressions that did not have a direct equivalent in Turkish were replaced with culturally appropriate terms. One significant challenge was the translation of idiomatic expressions and phrases that lacked a direct equivalent in Turkish. The translators, together with healthcare professionals, worked collaboratively to overcome these linguistic and cultural differences. For example, idiomatic phrases were either adapted or replaced with alternative expressions that conveyed the same meaning in a culturally relevant way. Similarly, some medical terms were adjusted to ensure they were both accurate and understandable for Turkish-speaking patients. These adjustments were made in consultation with healthcare professionals, ensuring the terms were culturally appropriate without compromising medical accuracy. After the translation and back-translation steps were completed, the final version of the scale was shared with 10 experts (five nurses and five doctors) to gather feedback on its linguistic adaptation and comprehensibility. Based on the experts’ feedback, minor revisions were made to improve clarity and ensure that the items were both medically accurate and culturally appropriate. These revisions included changing verbs and selecting different words with the same meaning to enhance clarity. To further validate the comprehensibility of the scale, a pilot test was conducted with 35 patients, none of whom were included in the final sample. The pilot test revealed a few areas where the scale’s items were unclear or difficult for patients to understand. For instance, certain medical phrases were found to be too complex or unfamiliar. Based on the feedback from the pilot test, additional adjustments were made, such as simplifying the language and rewording complex phrases. This final round of revisions ensured that the scale was fully understandable to Turkish-speaking patients, making it both linguistically and culturally appropriate. The Content Validity Index (CVI), a widely accepted measure of content validity, was calculated for the scale. To assess its content validity, feedback was collected from 12 experts who rated the items as “appropriate”, “needs revision”, or “not appropriate”. Based on their evaluations, the Content Validity Ratio (CVR) and Content Validity Index (CVI) for each item were calculated following established guidelines. The feedback was collected in three categories: “appropriate”, “needs revision”, and “not appropriate”. These categories were used to assess whether each item measured the intended attribute of the scale: “Appropriate” indicated that the item adequately measured the intended attribute of the scale. “Needs revision” meant that the item required revisions to better measure the intended attribute. The necessary corrections were made for the items placed in this category. “Not appropriate” indicated that the item was not suitable for the scale and did not measure the intended attribute effectively. There were no items placed in this category. The Content Validity Ratio (CVR) was calculated based on the number of experts who rated each item as “appropriate”. This calculation provided the CVR values, which were then used to determine the overall content validity of the scale [20,21,22,23].

Content Validity Ratio (CVR) for each item: (Number of experts who rated the item as “appropriate”)/(Total number of experts)/2 (−1);

Content Validity Index (CVI): (Sum of all the CVRs for each item)/(Total number of items).

### 2.3. Participants and Setting

Data for the study were collected between July 2024 and January 2025 from a diabetes education clinic in Türkiye, as well as through an online form distributed via a social media platform where the researchers shared diabetes-related information with the public. In validity and reliability studies of scales, it is recommended that the sample size be at least ten times the number of scale items [19]. Additionally, Comrey and Lee (1992) recommend the following sample sizes for factor analysis: 50 (very poor), 100 (poor), 200 (fair), 300 (good), 500 (very good), and 1000 (excellent) [24,25]. Therefore, a minimum of 500 individuals was targeted, and a total of 623 participants was reached. A test–retest was administered to the first 200 participants four weeks after the initial data collection. The study included participants aged 65 years or older who had been diagnosed with Type 2 diabetes, were using insulin therapy (either alone or in combination with oral antidiabetic drugs), and had experienced at least one hypoglycemic episode in the past six months. Individuals were excluded if they had neurological or mental disorders, visual or hearing impairments, were unable to communicate in Turkish, or were unwilling to participate (self-reported).

### 2.4. Data Collection Form

#### 2.4.1. Diabetes Identification Form

This form consists of questions about the sociodemographic characteristics of people with diabetes, such as age, gender, and education level, as well as their diabetes history, including details such as the type of diabetes, the date of diagnosis, and the frequency of hypoglycemic episodes over the past six months [4,8,16,26]. Data regarding the frequency of hypoglycemia were collected by asking participants the question, “How many hypoglycemic episodes have you experienced in the last six months?” A hypoglycemic episode was defined as an event during which the participant experienced typical symptoms of hypoglycemia and confirmed the episode with a self-monitored blood glucose measurement below 70 mg/dL using a personal glucometer. Only episodes that met both the symptomatic and biochemical criteria were included. These data were based on self-reported values and were not verified by healthcare professionals or medical records.

#### 2.4.2. Hypoglycemia Problem-Solving Scale (HPSS)

The Hypoglycemia Problem-Solving Scale (HPSS) is a 24-item tool designed to assess the problem-solving abilities of individuals with diabetes in managing hypoglycemia. The scale includes seven factors: problem-solving perception, detection control, identifying problem attributes, setting goals, seeking preventive strategies, evaluating strategies, and immediate management. The HPSS was validated using Exploratory Factor Analysis and demonstrated good internal consistency, with a Cronbach’s alpha of 0.83. This tool aims to support diabetes self-management by evaluating and enhancing patients’ problem-solving responses to hypoglycemic episodes. The scale uses a 0–4 Likert-type scoring system, with total scores ranging from 0 to 96. While the scale does not have a predefined cut-off point, higher scores indicate better problem-solving abilities for managing hypoglycemia. This scoring reflects an individual’s capacity to address and resolve challenges related to hypoglycemia, with higher scores signifying more effective problem-solving skills [15]. The Hypoglycemia Problem-Solving Scale (both English and Turkish versions) is provided in the Supplementary Materials (Table S1).

### 2.5. Statistical Analysis

In this study, data analysis was conducted using SPSS 22.0 and AMOS 25.0. A 95% confidence interval (CI) and a significance level of *p* < 0.001 were applied. The following statistical procedures were conducted [19,20,21,22,23,24,25,27,28]:

- Descriptive Statistics: Descriptive statistics were utilized to analyse the distribution of sociodemographic characteristics among older adults with diabetes. Additionally, mean and standard deviation (SD) values were calculated for the total scale and its subdimensions to summarize the overall scoring patterns.
- Language Validity: The scale underwent translation and back-translation procedures, followed by expert feedback from 10 specialists. The agreement among experts was evaluated using Kendall’s W test. A pre-test of the scale was conducted with participants.
- Content Validity: To assess content validity, Lawshe’s technique was employed, and both the Content Validity Ratio (CVR) and Content Validity Index (CVI) were calculated based on expert evaluations.
- Sample Adequacy: The adequacy of the sample for factor analysis was evaluated using the Kaiser–Meyer–Olkin (KMO) test.
- Data Suitability for Factor Analysis: The appropriateness of the data for factor analysis was evaluated using Bartlett’s test of sphericity. Inter-item correlations were reviewed, and no barriers against factor analysis were identified.
- Construct Validity: Exploratory Factor Analysis (EFA): Conducted to explore the factor structure of the scale. For the validity analysis, the dataset was divided into two subsets: the first subset was used for Exploratory Factor Analysis (EFA), while the second subset was utilized for Confirmatory Factor Analysis (CFA). As part of the Exploratory Factor Analysis (EFA), Principal Component Analysis was used for factor extraction, and Varimax rotation with Kaiser Normalization was applied as the rotation method.
- Confirmatory Factor Analysis (CFA): Confirmatory Factor Analysis (CFA) was conducted to evaluate the model fit indices.
- Test–Retest Reliability: The scale was re-administered to the first 200 participants after four weeks, and test–retest correlations were calculated.
- Internal Consistency: Item analyses and item-total correlations were examined. Cronbach’s alpha coefficient was calculated to assess the reliability of internal consistency. These analyses comprehensively tested the scale’s validity and reliability properties [19,20,21,22,23,24,25,27,28].

### 2.6. Ethics

Written permission was obtained from the author who developed the scale. The Non-Interventional Research Ethics Committee approved the study. Permission was obtained from the hospital where the study was conducted. Written and verbal consent was obtained from diabetic individuals participating in the study. The study was conducted in compliance with the Declaration of Helsinki.

## 3. Results

### 3.1. Sample Characteristics

The study included 623 participants, with a mean age of 72 ± 5.5 years. Of the participants, 51.8% were female and 48.2% male. Education levels were distributed as 6.7% illiterate, 48.5% elementary school, 25.2% high school, and 19.6% university graduates. The mean duration of diagnosis of the participants was 13.31 ± 7.97 years. Treatment types included insulin (43.2%) and insulin+oral antidiabetic drugs (OAD) (56.8%). Hypoglycemia frequency in the last six months varied, with 48.2% reporting 1–5 episodes and 17.3% over 16 episodes (Table 1).

### 3.2. Language Validity and Content Validity

For language validity, expert evaluations demonstrated a high level of agreement on the scale items (Kendall’s W = 0.83, indicating strong agreement, *p* < 0.05). During the pilot study, individuals with diabetes confirmed that the scale items were clear and understandable. Based on their suggestions, some items (Items 4, 12, and 23) were revised to enhance clarity. Content validity was evaluated using the Content Validity Index (CVI), a widely recognized measure developed by Lawshe. To assess content validity, the Content Validity Ratio (CVR) for each item was calculated based on the Lawshe method. A CVR value of 0.56 is considered the threshold for statistical significance in establishing content validity. The individual CVRs for the scale items ranged from 0.83 to 1.00, indicating strong content validity for each item. The overall CVI, calculated across all items, was 0.97, further supporting the high content validity of the scale.

### 3.3. Construct Validity

**Figure 1:**
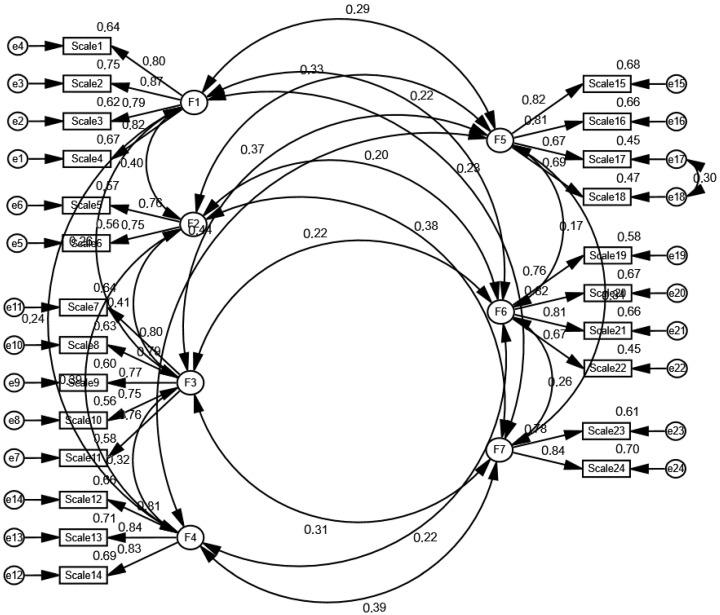
The relationship between the factors of HPSS.

The Kaiser–Meyer–Olkin (KMO) value for the study was 0.85, and Bartlett’s test of sphericity yielded χ^2^ = 7590.85 (*p* < 0.001), suggesting that the data were appropriate for factor analysis. In the Exploratory Factor Analysis (EFA), seven factors were extracted, accounting for 74.22% of the total variance (Table 2). In the Confirmatory Factor Analysis (CFA), the model fit indices were determined. The χ^2^/df value was 2.536, which is within the acceptable range. Additionally, the CFI (0.95), GFI (0.92), NFI (0.92), IFI (0.95), and TLI (0.94) indices were found to be within the generally accepted fit thresholds. The RMSEA value was 0.05, which is within the acceptable range of 0.05–0.08, indicating that the model’s fit level is acceptable. The RMR value was calculated as 0.02, and its low value suggests a low error level in the model. The standardized factor loadings and correlations between factors obtained from the CFA are shown in Figure 1.

### 3.4. Internal Consistency Analysis

For the test–retest reliability evaluation, Spearman’s rho correlation analysis was conducted, and the correlation coefficient between the first application and the application made four weeks later was found to be 0.99 (*p* < 0.001). This result indicates a very strong and statistically significant positive relationship between the two applications. The item-total correlation coefficient of the scale was found to be higher than 0.20, and the correlation coefficients were statistically significant. The lower and upper 27% of the sample were determined. It was also determined that there were significant differences between the lower and upper groups of the scale’s items (*p* < 0.001) (Table 3). The overall Cronbach’s alpha value of the scale is 0.88 (Table 4).

## 4. Discussion

**Figure 2:**
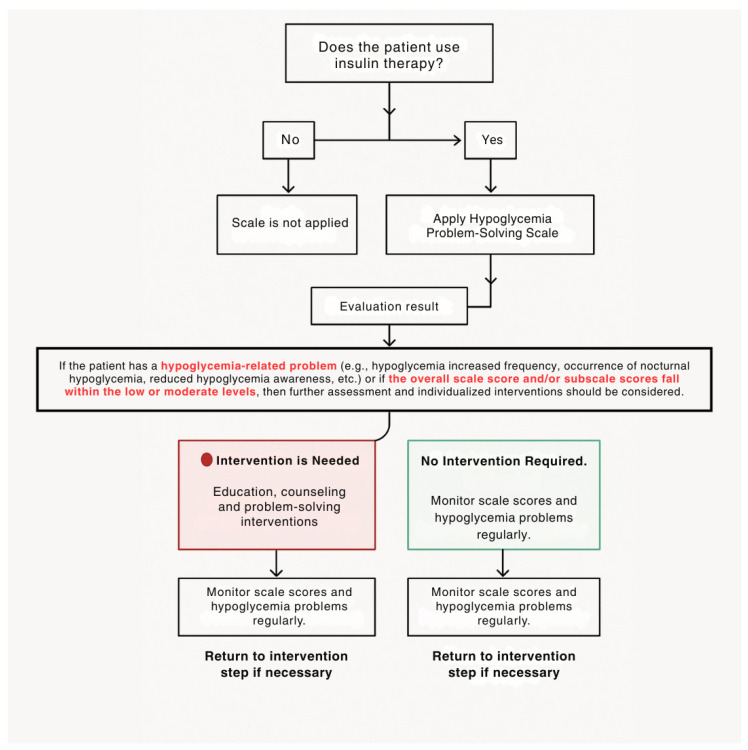
Hypoglycemia Problem-Solving Scale decision tree.

Hypoglycemia is a significant risk for diabetes management and can be particularly problematic in older adults [8,26]. The Hypoglycemia Problem-Solving Scale provides a detailed opportunity to assess the underlying causes of hypoglycemia, which is commonly experienced by individuals with diabetes. This scale can serve as a guide for healthcare professionals. The scale is suitable for use by healthcare professionals in clinical practice. It is valuable for guiding individualized patient education and counseling processes in older adults with diabetes. The subdimensions of the scale allow for a focused approach on the problematic areas related to hypoglycemia in individuals with diabetes. For example, it provides an opportunity to assess whether there are deficiencies in the individual’s hypoglycemia prevention strategies, a reduced ability to detect hypoglycemia, or an inability to provide immediate intervention. The scale allows for a detailed examination of such issues. Furthermore, it enables interventions that are specific to the problematic areas. Healthcare professionals may not always adequately prepare patients and their families to identify issues and develop preventive strategies before experiencing hypoglycemia. Such strategies can often lead to negative attitudes in patients and families rather than skill development. Therefore, the scale can help individuals with diabetes identify the causes of hypoglycemia, develop problem-solving strategies, and improve their ability to assess and address problems. These strategies also support the comfort of the individual’s family and caregivers [15,18]. In this study, although all participants had experienced at least one hypoglycemic event, this does not necessarily indicate that every individual required intervention. A history of hypoglycemia is essential for understanding and meaningfully responding to the items of the scale. Individuals who have never experienced hypoglycemia would not be able to accurately complete a scale that evaluates problem-solving skills specific to this condition. Therefore, only individuals with prior hypoglycemia experience were included in the study. The frequency of hypoglycemia among participants varied considerably; while some experienced episodes 2–3 times per week, others reported episodes once a month or once every three months. These differences reflect variations in individual risk levels and self-management needs. Any change in a patient’s hypoglycemia-related status (e.g., increased frequency, altered symptom threshold, occurrence of severe hypoglycemia, decreased awareness, etc.) should serve as a clinical alert for healthcare professionals. For example, if a patient experiences hypoglycemia once a month, this may be considered normal for an older adult using insulin and may not necessitate an educational intervention. However, it is important for the healthcare provider to assess whether such a change reflects a usual glycemic fluctuation or a new clinical issue. Furthermore, even if the individual’s condition remains stable, a decrease in the Hypoglycemia Problem-Solving Scale (HPSS) score may indicate an increased risk requiring intervention. In such cases, providing education and support would be appropriate, even in the absence of a newly emerged hypoglycemia problem. Conversely, if there are no new issues related to hypoglycemia and the scale score remains high, routine monitoring alone may be sufficient. The decision-tree guidelines for the use of the scale in clinical practice are shown in Figure 2. This study makes a significant contribution to the literature as it is the first scale in Turkish to assess hypoglycemia problem-solving skills in older adults. In the existing literature, only a version of the scale developed by Wu et al. is available. This section discusses the validity and reliability findings of the scale within the framework of the limited literature.

In cross-cultural scale-adaptation studies, the first step is ensuring linguistic validity [21,22]. The linguistic validity of the TR-HPSS was established through translation, back-translation, expert evaluation, and a pilot study conducted with patients. Regarding linguistic validity, expert evaluations indicated a high level of agreement on the scale items (Kendall’s W coefficient: 0.83, *p* < 0.05). Based on their suggestions, some items (Items 4, 12, and 23) were revised to enhance clarity. The administration of the TR-HPSS in the Turkish population takes approximately 5–10 min and is clear and comprehensible for older adults with diabetes. The scale can be used for individuals experiencing mild, moderate, and severe hypoglycemia. In the study by Wu et al., it was reported that the HPSS could be easily administered to individuals with diabetes experiencing hypoglycemia in less than 20 min [15]. To determine the low, moderate, and high-level score ranges for the total and subscale scores of the Hypoglycemia Problem-Solving Scale (HPSS), the expert opinion method was employed. For this purpose, qualified input was obtained from seven healthcare professionals experienced in hypoglycemia management (one endocrinologist, three diabetes nurses, and three academic nurses specialized in internal medicine nursing).

The process was carried out in four stages:

1. Selection of the Expert Panel: Experts were selected based on their clinical experience (≥5 years) and their involvement in patient follow-up or research related to hypoglycemia.
2. Evaluation of Scale Score Ranges: Open-ended questions were presented to the experts regarding which score ranges for the total score (0–96) and seven subscales of the HPSS would represent low, moderate, and high levels.
3. Collection of Opinions and Consensus Building: The number of experts who agreed on each proposed range was counted. Score ranges for which at least 85% of the experts (e.g., 6 out of 7) reached agreement were defined as “accepted levels”.
4. Final Score Classification: Score ranges with strong consensus among experts were included in the scale’s classification system (Table 5) [21,22,25,27,28,29].

Content validity was assessed using the Content Validity Index (CVI), a widely used measure developed by Lawshe. According to Lawshe’s method, a CVI value exceeding 0.56 for 12 experts is considered indicative of significant content validity [20,21,22]. In this study, the CVI was calculated as 0.97, confirming a high level of content validity for the scale. In Exploratory Factor Analysis (EFA), the Kaiser–Meyer–Olkin (KMO) test evaluates the adequacy of the sample size for factor analysis, with values greater than 0.60 deemed acceptable. Bartlett’s test of sphericity assesses whether significant correlations exist among the items in the scale [20,21,22,24,25,27,28]. In this study, the KMO value was 0.85, and Bartlett’s test of sphericity yielded χ^2^ = 7590.85 (*p* < 0.001), confirming the suitability of the data for factor analysis. The literature suggests that in multidimensional scales, an explained variance between 40% and 60% is generally considered sufficient [20,21,22,24,25,27,28]. In this study, exploratory factor analysis (EFA) revealed seven factors, which together explained 74.22% of the total variance, indicating a strong underlying factor structure. According to the EFA results, the factor loadings of the TR-HPSS exceeded 0.30. Factor loading represents the strength of the relationship between an item and its corresponding factor. The literature emphasizes that a minimum factor loading of 0.30 is required to determine the appropriate factor placement of items [21,22]. In the study by Wu et al., the original scale’s factor analysis confirmed a seven-factor structure with eigenvalues higher than 1. The scale was found to account for 73.14% of the explained variance. Among the seven factors, the problem-solving perception factor explained the majority of the variance (46.32%) [15]. In the Turkish HPSS study, Exploratory Factor Analysis (EFA) was performed on one half of the sample, while Confirmatory Factor Analysis (CFA) was conducted on the other half. The results of the CFA indicated that the χ^2^/df value was 2.536, which is within the acceptable range, suggesting a good fit for the model. Additionally, several fit indices were examined, all of which fell within the generally accepted thresholds for model fit. Specifically, the CFI (0.95), GFI (0.92), NFI (0.92), IFI (0.95), and TLI (0.94) values were all satisfactory, demonstrating that the model is well supported by the data. The results of both the EFA and CFA provide strong support for the structural validity of the TR-HPSS, demonstrating that it is a valid tool for assessing hypoglycemia problem-solving skills [21,22,25,27,28].

Test–retest reliability refers to the administration of the same scale to the same group at two different points in time to evaluate its stability. A correlation coefficient close to 1.00 indicates that the scale yields consistent results over time and has high reliability. The time interval between the two applications in the test–retest method is critical. It should be short enough to ensure that the attitudes and opinions of individuals with diabetes do not change, yet long enough to prevent them from recalling their previous responses. Therefore, a test–retest interval of 2 to 4 weeks is commonly recommended. To obtain more reliable results, it is also advised that the scale be administered by the same person and in the same setting [22]. In this study, the scale was administered in the same outpatient clinic room by the same researcher. It is known that the patients did not receive any additional medical consultation or diabetes education during the period between the first and second administrations of the scale. In this study, the Spearman’s rho correlation analysis conducted to evaluate test–retest reliability showed a correlation coefficient of 0.99 between the first application and the re-application after four weeks (*p* < 0.001). In the study by Wu et al., the test–retest reliability was assessed by having participants complete the scale again after two weeks. The correlation between participants’ responses was found to be 0.81 [15]. In the Turkish HPSS study, it was found that the item-total correlation coefficient was higher than 0.20, and the correlation coefficients were statistically significant. In the study by Wu et al., the item-total correlation values of the scale ranged between rho: 0.40 and 0.76 [15]. In the Turkish HPSS study, it was found that there were significant differences between the items’ lower and upper groups (*p* < 0.001). The literature indicates that a Cronbach’s alpha coefficient value between 0.60 and 0.80 is considered moderately reliable, while a value between 0.80 and 1.00 is considered highly reliable [21,22]. The overall Cronbach’s alpha value of the scale was 0.88, indicating high internal consistency and reliability. The Corrected Item-Total Correlation values were mostly above 0.40, indicating that the items are consistent with the overall scale. The changes in Cronbach’s alpha value when any item was removed were minimal (ranging from 0.875 to 0.880), suggesting that all items make a meaningful contribution to the scale and help maintain its consistency. In the study by Wu et al., the Cronbach’s alpha value of the scale was found to be 0.83. The Cronbach’s alpha values for the seven factors ranged from 0.70 to 0.86 [15]. These findings demonstrate that the HPSS is a valid and reliable measurement tool. The results indicate that the scale can reliably assess the hypoglycemia problem-solving abilities of Turkish older adults with diabetes.

### Limitations

The study is self-reported by individuals with diabetes. HbA1c data were not included in this study, as the primary aim was to evaluate the validity and reliability of the scale used to assess hypoglycemia problem-solving skills. The study was not designed to examine metabolic control or biochemical parameters. This is acknowledged as a limitation, and future studies aiming to explore the relationship between hypoglycemia problem-solving skills and glycemic control are encouraged to include objective clinical indicators such as HbA1c. In this study, the test–retest reliability was assessed with a four-week interval. Although this interval is consistent with the 2–4 week range recommended in the literature, the very high correlation coefficient obtained (r = 0.99) may be associated with potential memory effects. Participants did not receive additional education or change in their diabetes management during this period, but it is possible that some of them remembered the scale items and responded accordingly. This should be considered a limitation in the interpretation of the test–retest reliability findings.

## 5. Conclusions

In this study, the psychometric properties of the Turkish version of the HPSS were examined. The findings reveal that Turkish HPSS is a valid and reliable measurement tool for assessing hypoglycemia problem-solving abilities older adults with diabetes. The high levels of validity and reliability of Turkish HPSS demonstrate that it provides a reliable tool for evaluating individuals’ self-efficacy in managing hypoglycemia within diabetes management processes. Considering its potential to prevent complications related to hypoglycemia and develop individual coping strategies, Turkish HPSS is expected to contribute significantly to clinical practice and scientific research. It is anticipated that this scale could not only measure individuals’ hypoglycemia management skills but also serve as an important tool in assessing the effectiveness of innovative interventions aimed at diabetes management. The use of Turkish HPSS in Türkiye holds strategic importance for both healthcare professionals and policymakers in developing sustainable and individu-alized approaches to diabetes care. Therefore, the results of this study highlight the need for future research aimed at developing and testing tailored intervention strategies based on specific patterns of hypoglycemia experiences and problem-solving deficiencies. In addition, these findings also emphasize the importance of monitoring hypoglycemia-related problem-solving skills in individuals with diabetes. In cases where HPSS scores or hypoglycemia-related problems are identified, individualized interventions should be considered. However, determining which interventions are most effective for specific hypoglycemia-related issues (e.g., frequency, awareness, severity) or for improving HPSS levels is beyond the scope of the present study. Future studies may focus on developing tailored interventions specific to each subscale and type of problem. Future studies applying the scale to broader populations may provide a better understanding of cultural, demographic, and clinical differences in hypoglycemia management. In this context, the adoption of Turkish HPSS as a standard assessment tool for hypoglycemia management in Türkiye will not only improve individual health outcomes but also inform national health policies’ strategic goals focusing on hypoglycemia management. Thus, it will support the development of individualized approaches in diabetes management.

## Figures and Tables

**Table 1:** Descriptive characteristics of individuals with diabetes (*n = 623*).

Characteristics	Value
**Age** (Mean ± Std.)	72 ± 5.5
**Gender** *n* (%)	
Female	323 (51.8%)
Male	300 (48.2%)
**Education** *n* (%)	
Illiterate	42 (6.7%)
Elementary School	302 (48.5%)
High School	157 (25.2%)
University	122 (19.6%)
**Time of Diagnosis** Min–Max (Mean ± Std.)	1–39 (13.31 ± 7.97)
**Treatment** *n* (%)	
Insulin	269 (43.2%)
Insulin + OAD	354 (56.8%)
**Hypoglycemia Frequency in the Last 6 Months** *n* (%)	
1–5 times	300 (48.2%)
6–10 times	116 (18.6%)
11–15 times	99 (15.9%)
≥16 times	108 (17.3%)

OAD: oral antidiabetic drugs.

**Table 2:** Exploratory Factor Analysis (EFA) and factor loadings.

Factors	Item Number/Item Content	Factor Loading	Corrected Item-Total Correlation
**Problem-solving perception**	1R. When my attempt to prevent hypoglycaemia fails, I become discouraged and cannot think clearly.	0.83	0.47
	2R. The difficulty I encounter in preventing hypoglycaemia makes me feel depressed or angry.	0.87	0.45
	3R. I often worry about how to prevent hypoglycaemia but have not taken any action to address it.	0.83	0.45
	4R. When I cannot prevent hypoglycaemia I feel stupid.	0.83	0.47
**Detection control**	5. I know how to handle hypoglycaemia.	0.83	0.44
	6. I do not give up when my initial attempt to effectively prevent hypoglycaemia fails, and I believe that I will ultimately find the best approach to solve it.	0.81	0.42
**Identifying problem attributes**	7. When hypoglycaemia occurs, I examine for any event that may contribute to the occurrence of hypoglycaemia.	0.81	0.52
	8. When my efforts to prevent hypoglycaemia are ineffective, I return to where I made the mistakes and attempt other methods.	0.81	0.51
	9. When I am not satisfied with the results of preventing hypoglycaemia, I will find a better method and attempt it again.	0.79	0.50
	10. When my attempt to prevent hypoglycaemia fails, I will analyse and identify my mistake.	0.81	0.43
	11. To prevent hypoglycaemia, I attempt to learn as much information on the occurrence of hypoglycaemia as possible.	0.76	0.53
**Setting problem-solving goals**	12. When I attempt to manage hypoglycaemia, I remember all the goals that I have set.	0.83	0.48
	13. When attempting to prevent hypoglycaemia, I set a goal so that I know what I need to achieve.	0.85	0.46
	14. I will attempt to prevent hypoglycaemia and achieve all the goals I have set.	0.85	0.48
**Seeking preventive strategies**	15. I usually speak with my family when I am attempting to prevent hypoglycaemia.	0.78	0.50
	16. I speak with health professionals when hypoglycaemia prevention becomes complex and difficult.	0.79	0.48
	17. When hypoglycaemia prevention becomes complex and difficult, I seek help from friends or pay close attention to my physical changes.	0.79	0.47
	18. When hypoglycaemia prevention becomes complex and difficult, I learn how to prevent hypoglycaemia from people who have the same problem as mine.	0.82	0.46
**Evaluating strategies**	19. After implementing the method for hypoglycaemia prevention, I evaluate the effectiveness of this method in preventing hypoglycaemia.	0.82	0.37
	20. When preventing hypoglycaemia, I attempt my own method to increase the chance of success.	0.86	0.34
	21. When determining the best hypoglycaemia prevention method, I attempt to predict the possible outcome.	0.85	0.35
	22. I understand hypoglycaemia prevention is one of the problems that must be resolved in diabetic care.	0.70	0.45
**Immediate management**	23R. When I experience hypoglycaemia, I usually snack, stop all activity, or stop insulin injections, and do not think about prevention.	0.86	0.41
	24R. To me, hypoglycaemia is an easily manageable problem and does not need to be a concern.	0.86	0.42
**Total variance:** 74.22%			

Extraction method: Principal Component Analysis. Rotation method: Varimax with Kaiser Normalization. R: reverse encoded.

**Table 3:** Difference between the lower and upper groups of HPSS.

Items	Groups	n	Mean	Standard Deviation	5% Confidence Interval Lower–Upper	*t*	df	*p*
Item 1	Lower Group	169	1.20	0.82	[−0.91–0.60]	−9.67	303.96	<0.001
	Upper Group	169	1.95	0.59				
Item 2	Lower Group	169	1.22	0.82	[−0.95–0.64]	−9.94	318.05	<0.001
	Upper Group	169	2.02	0.64				
Item 3	Lower Group	169	1.22	0.82	[−0.81–0.52]	−8.92	285.14	<0.001
	Upper Group	169	1.89	0.52				
Item 4	Lower Group	169	1.24	0.85	[−0.87–0.56]	−9.05	295.37	<0.001
	Upper Group	169	1.96	0.57				
Item 5	Lower Group	169	1.33	0.8	[−0.67–0.37]	−6.83	303.24	<0.001
	Upper Group	169	1.85	0.57				
Item 6	Lower Group	169	1.30	0.76	[−0.67–0.37]	−7.02	320.09	<0.001
	Upper Group	169	1.82	0.6				
Item 7	Lower Group	169	1.13	0.67	[−0.95–0.69]	−12.4	318.19	<0.001
	Upper Group	169	1.95	0.53				
Item 8	Lower Group	169	1.15	0.67	[−1.02–0.76]	−13.76	313.06	<0.001
	Upper Group	169	2.04	0.5				
Item 9	Lower Group	169	1.19	0.7	[−0.95–0.68]	−12.17	312.38	<0.001
	Upper Group	169	2.01	0.52				
Item 10	Lower Group	169	1.26	0.66	[−0.83–0.56]	−10.36	327.11	<0.001
	Upper Group	169	1.96	0.56				
Item 11	Lower Group	169	1.16	0.71	[−0.95–0.68]	−11.82	317.25	<0.001
	Upper Group	169	1.98	0.55				
Item 12	Lower Group	169	1.28	0.8	[−0.84–0.54]	−9.11	307.45	<0.001
	Upper Group	169	1.98	0.58				
Item 13	Lower Group	169	1.23	0.8	[−0.84–0.53]	−8.9	314.05	<0.001
	Upper Group	169	1.92	0.61				
Item 14	Lower Group	169	1.20	0.76	[−0.87–0.58]	−9.98	309.52	<0.001
	Upper Group	169	1.93	0.56				
Item 15	Lower Group	169	1.11	0.75	[−0.98–0.69]	−11.03	328.26	<0.001
	Upper Group	169	1.95	0.64				
Item 16	Lower Group	169	1.13	0.67	[−0.95–0.68]	−11.94	330.62	<0.001
	Upper Group	169	1.95	0.59				
Item 17	Lower Group	169	1.21	0.74	[−0.90–0.61]	−10.29	325.23	<0.001
	Upper Group	169	1.97	0.61				
Item 18	Lower Group	169	1.19	0.76	[−0.93–0.63]	−10.12	328.67	<0.001
	Upper Group	169	1.98	0.55				
Item 19	Lower Group	169	1.32	0.73	[−0.71–0.43]	−7.93	319.41	<0.001
	Upper Group	169	1.89	0.58				
Item 20	Lower Group	169	1.37	0.8	[−0.80–0.49]	−8.39	310.48	<0.001
	Upper Group	169	2.02	0.61				
Item 21	Lower Group	169	1.34	0.8	[−0.75–0.44]	−7.57	319.752	<0.001
	Upper Group	169	1.94	0.63				
Item 22	Lower Group	169	1.21	0.69	[−0.90–0.63]	−11.06	326.525	<0.001
	Upper Group	169	1.98	0.58				
Item 23	Lower Group	169	1.31	0.83	[−0.80–0.48]	−7.9	315.922	<0.001
	Upper Group	169	1.95	0.64				
Item 24	Lower Group	169	1.27	0.79	[−0.78–0.49]	−8.53	303.8	<0.001
	Upper Group	169	1.91	0.56				

*p* < 0.001, df: degrees of freedom, *t*: *t* test.

**Table 4:** Reliability analysis.

Item—Total Statistics			
Items	Scale Mean If Item Deleted	Scale Variance If Item Deleted	Cronbach’s Alpha If Item Deleted
Item 1	37.87	68.91	0.87
Item 2	37.83	69.01	0.87
Item 3	37.85	69.35	0.87
Item 4	37.83	69.08	0.87
Item 5	37.83	69.65	0.87
Item 6	37.85	70.02	0.87
Item 7	37.89	69.00	0.87
Item 8	37.85	68.98	0.87
Item 9	37.82	69.12	0.87
Item 10	37.83	70.08	0.87
Item 11	37.83	68.69	0.87
Item 12	37.82	68.97	0.87
Item 13	37.82	69.13	0.87
Item 14	37.83	69.11	0.87
Item 15	37.87	68.72	0.87
Item 16	37.85	69.25	0.87
Item 17	37.83	69.07	0.87
Item 18	37.83	69.07	0.87
Item 19	37.82	70.49	0.87
Item 20	37.78	70.51	0.88
Item 21	37.80	70.59	0.88
Item 22	37.84	69.83	0.87
Item 23	37.78	69.61	0.87
Item 24	37.84	69.76	0.87
**Total Cronbach’s Alpha: 0.88**			

**Table 5:** HPSS scoring.

HPSS	Low Level	Moderate Level	High Level
**HPSS Total**	**≤49**	**50–79**	**80–96**
**HPSS Subscales**			
Problem-Solving Perception	0–5	6–11	12–16
Detection Control	0–3	4–6	7–8
Identifying Problem Attributes	0–7	8–14	15–20
Setting Problem-Solving Goals	0–4	5–8	9–12
Seeking Preventive Strategies	0–5	6–11	12–16
Evaluating Strategies	0–5	6–11	12–16
Immediate Management	0–3	4–6	7–8

HPSS; Hypoglycemia Problem-Solving Scale.

## Data Availability

The raw data supporting the conclusions of this article will be made available by the authors on request.

## Supplementary Materials

The following supporting information can be downloaded at: https://www.mdpi.com/article/10.3390/healthcare13090997/s1, Table S1: Turkish Hypoglycemia Problem-Solving Scale (English and Turkish verson).
