# Supplementary material for: A Psychometric Evaluation of the Hypoglycemia Problem-Solving Scale (HPSS) in Turkish Older Adults with Diabetes
Source: Healthcare (Basel). 2025 Apr 25;13(9):997. doi: 10.3390/healthcare13090997 (PMC12071617; doi:10.3390/healthcare13090997)
Supplement: Supplementary file 1 [file healthcare-13-00997-s001.zip › healthcare-3559939-supplementary.pdf]

Supplement

Table S1. Hipoglisemi Problem Çözme Ölçeği (TR-HPSS)

Ölçek Maddeleri	Bana hiç uygun değil	Bana nadiren uygun	Bana bazen uygun	Bana çoğu zaman uygun	Bana tamamen uygun
**1*.**Hipoglisemiyi önleme çabam başarısız olduğunda cesaretim kırılır ve net düşünemem.	0	1	2	3	4
**2*.**Hipoglisemiyi önlerken karşılaştığım zorluklar beni üzgün veya sinirli hissettirir.	0	1	2	3	4
**3*.**Hipoglisemiyi nasıl önleyeceğim konusunda sık sık endişelenirim, ancak bu konuda harekete geçmem.	0	1	2	3	4
**4*.**Hipoglisemiyi önleyemediğimde kendimi yetersiz hissederim.	0	1	2	3	4
**5**. Hipoglisemiyi nasıl yöneteceğimi bilirim.	0	1	2	3	4
**6.**Hipoglisemiyi etkili bir şekilde önleme çabam başarısız olsa bile pes etmem ve sonunda en iyi yöntemi bulacağıma inanırım.	0	1	2	3	4
**7.**Hipoglisemi yaşadığımda bu duruma neden olabilecek olayları gözden geçiririm.	0	1	2	3	4
**8.**Hipoglisemiyi önleme çabalarım başarısız olduğunda nerede hata yaptığımı gözden geçirir ve farklı yöntemler denerim.	0	1	2	3	4
**9.**Hipoglisemiyi önleme çabalarımın sonucundan memnun kalmazsam daha iyi bir yöntem bulup tekrar denerim.	0	1	2	3	4
**10.**Hipoglisemiyi önleme çabam başarısız olduğunda hatalarımı analiz eder ve nedenlerini belirlerim.	0	1	2	3	4
**11.**Hipoglisemiyi önlemek için hipogliseminin oluşumu hakkında olabildiğince fazla bilgi edinmeye çalışırım.	0	1	2	3	4
**12.**Hipoglisemiyi yönetmeye çalışırken belirlediğim tüm hedefleri hatırlarım.	0	1	2	3	4
**13.**Hipoglisemiyi önlerken neyi başarmam gerektiğini bilmek için hedefler koyarım.	0	1	2	3	4
**14.** Hipoglisemiyi önlemek ve belirlediğim tüm hedeflere ulaşmak için çaba gösteririm.	0	1	2	3	4
**15.** Hipoglisemiyi önlemeye çalışırken genellikle ailemle konuşurum.	0	1	2	3	4
**16.** Hipoglisemi önlemek karmaşık ve zor hale geldiğinde sağlık profesyonellerine danışırım.	0	1	2	3	4
**17.** Hipoglisemiyi önlemek karmaşık ve zor hale geldiğinde arkadaşlarımdan yardım ister veya vücudumdaki değişikliklere dikkat ederim.	0	1	2	3	4
**18.** Hipoglisemi önlemenin karmaşık ve zor olduğu durumlarda, aynı sorunu yaşayan kişilerden bilgi ve deneyim edinirim.	0	1	2	3	4
**19.** Hipoglisemi önleme yöntemini uyguladıktan sonra bu yöntemin etkinliğini değerlendiririm.	0	1	2	3	4

**20.** Hipoglisemiyi önlerken başarı şansımı artırmak için kendi yöntemimi denerim.	0	1	2	3	4
**21.** En iyi hipoglisemi önleme yöntemini belirlerken olası sonucu tahmin etmeye çalışırım.	0	1	2	3	4
**22.** Hipoglisemi önlemenin diyabet bakımında çözülmesi gereken bir sorun olduğunu anlarım.	0	1	2	3	4
**23*.** Hipoglisemi yaşadığımda genellikle bir şeyler yer, tüm aktivitelerimi durdurur veya insülin enjeksiyonlarını keserim ve önleme hakkında düşünmem.	0	1	2	3	4
**24*.** Hipoglisemi benim için kolayca yönetilebilecek bir sorundur ve endişelenecek bir durum değildir.	0	1	2	3	4

*Madde ters kodlanır.

Table S1. Hypoglycemia Problem-Solving Scale (HPSS) (English Translation) [15].

	Item	Not at all true of me	Slightly true of me	Moderately true of me	Very true of me	Extremely true of me
1	When my attempt to prevent hypoglycaemia fails, I become discouraged and cannot think clearly. (R)	0	1	2	3	4
2	The difficulty I encounter in preventing hypoglycaemia makes me feel depressed or angry. (R)	0	1	2	3	4
3	I often worry about how to prevent hypoglycaemia but have not taken any action to address it. (R)	0	1	2	3	4
4	When I cannot prevent hypoglycaemia, I feel stupid. (R)	0	1	2	3	4
5	I know how to handle hypoglycaemia.	0	1	2	3	4
6	I do not give up when my initial attempt to effectively prevent hypoglycaemia fails, and I believe I will ultimately find the best approach to solve it.	0	1	2	3	4
7	When hypoglycaemia occurs, I examine for any event that may contribute to the occurrence of hypoglycaemia.	0	1	2	3	4
8	When my efforts to prevent hypoglycaemia are ineffective, I return to where I made the mistakes and attempt other methods.	0	1	2	3	4
9	When I am not satisfied with the results of preventing hypoglycaemia, I will find a better method and attempt it again.	0	1	2	3	4
10	When my attempt to prevent hypoglycaemia fails, I will analyze and identify my mistake.	0	1	2	3	4
11	To prevent hypoglycaemia, I attempt to learn as much information on the occurrence of hypoglycaemia as possible.	0	1	2	3	4
12	When I attempt to manage hypoglycaemia, I remember all the goals that I have set.	0	1	2	3	4
13	When attempting to prevent hypoglycaemia, I set a goal so that I know what I need to achieve.	0	1	2	3	4

14	I will attempt to prevent hypoglycaemia and achieve all the goals I have set.	0	1	2	3	4
15	I usually speak with my family when I am attempting to prevent hypoglycaemia.	0	1	2	3	4
16	I speak with health professionals when hypoglycaemia prevention becomes complex and difficult.	0	1	2	3	4
17	When hypoglycaemia prevention becomes complex and difficult, I seek help from friends or pay close attention to my physical changes.	0	1	2	3	4
18	When hypoglycaemia prevention becomes complex and difficult, I learn how to prevent hypoglycaemia from people who have the same problem as mine.	0	1	2	3	4
19	After implementing the method for hypoglycaemia prevention, I evaluate the effectiveness of this method in preventing hypoglycaemia.	0	1	2	3	4
20	When preventing hypoglycaemia, I attempt my own method to increase the chance of success.	0	1	2	3	4
21	When determining the best hypoglycaemia prevention method, I attempt to predict the possible outcome.	0	1	2	3	4
22	I understand hypoglycaemia prevention is one of the problems that must be resolved in diabetic care.	0	1	2	3	4
23	When I experience hypoglycaemia, I usually snack, stop all activity, or stop insulin injections, and do not think about prevention. (R)	0	1	2	3	4
24	To me, hypoglycaemia is an easily manageable problem and does not need to be a concern. (R)	0	1	2	3	4
